# The impact of antibiotic stewardship interventions and patient related factors on antibiotic prescribing in a vascular surgical department

**DOI:** 10.1007/s15010-023-02056-1

**Published:** 2023-06-08

**Authors:** M. M. Gruber, A. Weber, J. Jung, A. Strehlau, N. Tsilimparis, R. Draenert

**Affiliations:** 1 Stabsstelle Antibiotic Stewardship, LMU University Hospital, LMU Munich, Munich, Germany; 2 Hospital Pharmacy, LMU University Hospital, LMU Munich, Munich, Germany; 3 Max von Pettenkofer Institute, Faculty of Medicine, LMU Munich, Munich, Germany; 4 Division of Vascular Surgery, LMU University Hospital, LMU Munich, Munich, Germany

**Keywords:** Antibiotic stewardship, Vascular surgery, Comorbidity, Patient characteristics

## Abstract

**Purpose:**

The development of guidelines tailored to the departments’ needs and counselling during ward rounds are important antibiotic stewardship (AS) strategies. The aim was to analyse the impact of AS ward rounds and institutional guidelines as well as patient-related factors on antibiotic use in vascular surgical patients.

**Methods:**

A retrospective prescribing-analysis of 3 months (P1, P2) before and after implementing weekly AS ward rounds and antimicrobial treatment guidelines was performed. Choice of systemic antibiotics, days of antibiotic therapy and clinical data were obtained from electronic patient records.

**Results:**

During P2, the overall antibiotic consumption as well as the use of last-resort compounds like linezolid and fluoroquinolones decreased distinctly (overall: 47.0 days of therapy (DOT)/100 patient days (PD) vs. 35.3 DOT/100PD, linezolid: 3.7 DOT/100PD vs. 1.0 DOT/100PD, fluoroquinolones: 7.0 DOT/100PD vs. 3.2 DOT/100PD) while narrow-spectrum beta-lactams increased by 48.4%. Courses of antibiotics were de-escalated more often during P2 (30.5% vs. 12.1%, *p* = 0.011). Only in P2, an antibiotic therapy was initiated in patients suffering from more comorbidities (i.e. higher Charlson Comorbidity Index) more frequently. Other patient factors had no distinct impact on antibiotic prescribing.

**Conclusion:**

Weekly AS ward rounds improved adherence to institutional antibiotic treatment guidelines and antibiotic prescribing in vascular surgical patients. Clear patient-related determinants affecting choice of antibiotic therapies could not be identified.

**Supplementary Information:**

The online version contains supplementary material available at 10.1007/s15010-023-02056-1.

## Introduction

Antimicrobial stewardship (AS) is one possibility of fighting antimicrobial resistance—a global threat public health is facing more than ever: Murray et al. estimated in their study 4.95 million deaths associated with bacterial antimicrobial resistance worldwide in 2019, with 1.27 million attributable deaths [1]. Although the term antimicrobial stewardship is widely used, there is no clear definition of what antimicrobial stewardship exactly stands for. Dyar et al. defined antimicrobial stewardship as “a coherent set of actions which promote using antimicrobials responsibly” [2]. Still, AS strategies can vary highly between different AS programs. They include, among others, education and distribution of educational material or reminders as posters, the development of guidelines for antimicrobial use, audit and feedback for antibiotic prescribers or restrictive actions like special release for last-resort compounds with mandatory consultation of an infectious disease specialist and restricted formulary [2, 3]. AS interventions in general improve antibiotic prescribing without negatively affecting patient outcomes—often leading to a shorter hospital stay [3]. There are various factors with impact on achieving appropriate antibiotic prescribing [4–8]. It might depend on the setting (e.g. outpatient, inpatient, long-term care facilities), the study population (e.g. elderly patients, patients with respiratory tract infections only) or the perspective of how antibiotic prescribing can be influenced (e.g. prescriber, patient characteristics). Knowing whether and which patient factors are associated with physicians prescribing antibiotics inappropriately could help optimize antibiotic prescribing in the future. These factors could then be considered more intensively in future AS interventions allowing patients to benefit from individualized guidelines or other tailored AS strategies.

In the present study, we aimed to analyse the impact of AS ward rounds after the implementation of antimicrobial treatment guidelines on the overall antibiotic use in a vascular surgery department as, to the best of our knowledge, there is a paucity of data for the special group of vascular surgical patients. Furthermore, we concentrated on patient-related determinants influencing inpatient antibiotic prescribing and guideline adherence to the institutional guidelines in vascular surgical patients. On the basis of these findings, future AS interventions could be adapted for individual patient groups and further improve antibiotic prescribing.

## Methods

### Study population and setting

This monocentric observational study was conducted at the vascular surgery department of the university hospital LMU Munich. The LMU hospital—a 2.000-bed tertiary care hospital—serves around 500.000 patients a year with a catchment area of whole southern Germany for many specialties. The vascular surgical ward of the university hospital LMU comprises about 20 beds. All patients aged 18 years or older who were admitted to the general ward of the vascular surgery department from September 2018 through November 2018 (P1) and from September 2019 through November 2019 (P2) were included in the study. Patients with an incomplete set of data were excluded. In addition, patients with more than 50% of their hospital stay outside of the observation period were excluded due to a possible bias of the results, as a distinct part of the hospital stay did not contribute to the observation period. Two independent retrospective prescribing analyses of 3 months each, before (= P1) and after (= P2) implementing AS interventions, were carried out. The study was approved by the ethics committee of the university hospital LMU Munich (register-number 19-906).

### AS interventions

The AS program of the university hospital LMU Munich, composed of an infectious disease (ID) physician, an ID pharmacist and a clinical microbiologist, introduced weekly AS ward rounds at the general ward of the vascular surgery department in 2019. The AS ward rounds were based on an audit and feedback policy in cooperation with the current ward physician. Every patient on antimicrobial therapy was discussed by the surgeon and the AS team. The ward physician could, furthermore, present patients during the ward round who were currently not on antimicrobial therapy, but were suspected to have an infectious process. Antimicrobial therapy was reviewed regarding choice of substance, dosing, (de-) escalation opportunities and treatment duration. Particularly, recommendations about (de-)escalation of antibiotic therapy were made according to microbiological results. Furthermore, antimicrobial treatment guidelines issued in collaboration with the department of vascular surgery, hospital hygiene and medicinal microbiology were introduced in an educational session and then available in the hospital’s intranet (Fig. SM1, appendix). Additionally, the antimicrobialtreatment guidelines printed as pocket-cards were distributed among the physicians of the vascular surgery department.

### Data acquisition

For a factor model analysing the influence of patient related determinants on antibiotic prescribing, we included: age, sex, length of hospital stay, admission from a medical institution, in-hospital-mortality, rate of readmission after 30 days for infection, charlson comorbidity index (CCMI), intensive care stay, severity of vascular disease, penicillin allergy, foreign material in situ, glomerular filtration rate, immunosuppression, surgery, revision surgery. All relevant demographic and clinical data, choice of systemic antibiotics and days of antibiotic therapy (DOT) were manually obtained from electronic patient records and irreversibly anonymised after data collection.

### Definitions

**Fig. 1:**
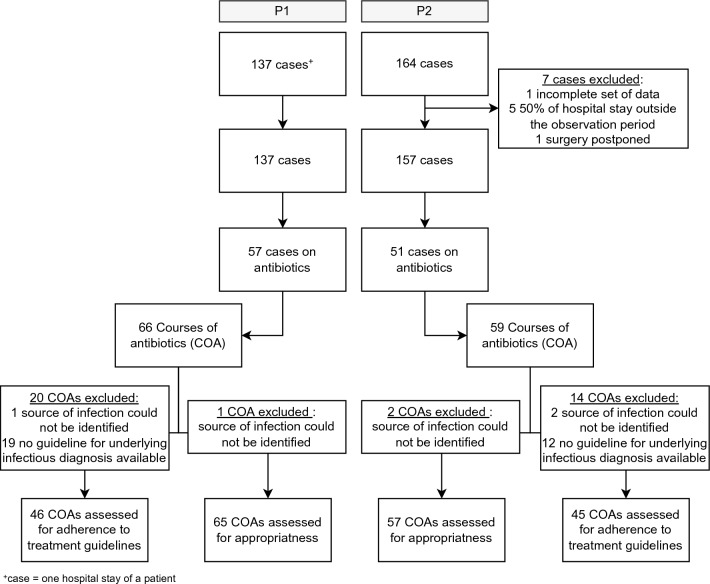
Flow chart of the included cases and courses of antibiotics (COA)

In this study, the analyses were based either on cases (the hospital stay of one patient), or courses of antibiotics (COA). Several cases could be assigned to one patient during the two study periods, if the patient was admitted repeatedly during the study period. One course of antibiotics was defined as continuous days of antibiotic treatment. After discontinuation of antibiotic treatment for more than 1 day, a new COA was determined, also within the same case (Fig. 1).

Antibiotics that were prescribed during the study periods were ranked according to their activity against drug-resistant bacteria [9–11] (Table 1). A change of antibiotic agent(s) during one COA could be classified as de-escalation or escalation as described previously [9, 11, 12]: De-escalation of antibiotic therapy was defined as a change of one or more antibiotics to an antibiotic of lower rank or termination of one or more antibiotics in a combination. However, if there was a restricted antibiotic (e.g. linezolid, tigecycline) continued after the change, it was not classified as de-escalation. A change of one or more antibiotics to an antibiotic of higher rank or starting an additional antibiotic to the existing antibiotic therapy was defined as escalation.

**Table 1:** Antibiotic ranking [9, 10]

Rank 1 (narrow spectrum)	Narrow spectrum penicillins, first- and second-generation cephalosporins, co-trimoxazole, doxycycline, oral fosfomycin, metronidazole
Rank 2 (broad spectrum)	Aminopenicillin/beta-lactamase inhibitor, third-generation cephalosporins, fluoroquinolones, macrolides, clindamycin, intravenous fosfomycin, rifampicin
Rank 3 (extended spectrum)	Fourth-generation cephalosporines, carbapenems, piperacillin/tazobactam, vancomycin
Rank 4 (restricted)	Linezolid, tigecycline

COAs were also evaluated according the choice of antibiotic agent. It could be classified as appropriate or inappropriate and further if it was in adherence with the local treatment guidelines or not. Guideline adherence regarding antibiotic substance was only evaluated if a current guideline for the underlying infectious diagnosis was available. Antibiotic substance was considered appropriate, unless at least one of the following criteria was of relevance:

- No evidence of infection according to the electronic file
- The expected spectrum of bacteria was not covered with the prescribed antibiotics
- The prescribed antibiotic(s) distinctly exceeded the expected spectrum of bacteria for the treated infection
- Prophylaxis pre- or post-surgery if not recommended by guidelines

If the source of infection could not be identified for a COA, they were not considered for statistical analysis. Each COA was retrospectively evaluated by an ID physician and a pharmacist for changes in antibiotic therapy and choice of antibiotic agent.

Severity of the underlying vascular disease (e.g. aortic aneurysm) was retrospectively analysed by consulting reference literature [13]. However, for some disorders there was no official classification available. In these cases (e.g. carotid body tumor) no clear classification could be made.

Antibiotic consumption was quantified in days of antibiotic therapy per 100 patient days (DOT/100PD). Length of antibiotic therapy (LOT) was defined as the total of in-hospital days of antibiotic therapy for one patient case.

### Outcomes and statistical analysis

Due to the small sample size in our study, the variables contributing to the identification of patient-related determinants that might influence antibiotic prescribing are analysed descriptively. The impact of weekly AS ward rounds together with antimicrobial treatment guidelines was assessed by comparing antibiotic consumption and changes in antibiotic therapy.

All categorial variables are presented as numbers with frequencies. To compare the categorial variables, χ^2^-test or Fisher ´s exact test was used. Continuous variables are shown as median with range while for the comparisons the Mann–Whitney U test was used. The statistical analysis was performed with IBM SPSS Statistics 26. Statistical significance was defined as *p* < 0.05.

## Results

### Patient characteristics

Over the two study periods, 137 (P1) and 164 (P2) patient cases were identified, respectively. In P2, seven cases had to be excluded. One patient had an incomplete set of data, another patient’s surgery was postponed and the patient was discharged after only 1 day. Five patients with more than 50% of their hospital stay outside of the observation period were further excluded. Thus, 137 (P1) and 157 (P2) patient cases were included in the statistical analysis (Fig. 1). In general, the two study groups were comparable (Table 2). However, more patients were admitted to the intensive or intermediate care unit during P2 (63.7) compared to P1 (51.8%) which was statistically significant (*p* = 0.04). Overall, the infectious diseases diagnoses were comparable for both study periods (Table 3). During P2, however, there were more diagnoses of pneumonia (7.6% (P1) vs. 20.3% (P2), *p* = 0.038). In contrast, more COAs with patients treated for urinary tract infections were observed in P1 (21.2% (P1) vs. 10.2% (P2), *p* = 0.093). The spectrum of underlying vascular diagnoses did not differ between the two study periods.

**Table 2:** Patient characteristics comparing P1 and P2

Characteristics	P1 (n = 137)	P2 (n = 157)	*p*-value
Age—years, median (range)	69.0 (30–91)	70.0 (22–97)	0.408
Sex, female—no. (%)	41 (29.9)	40 (25.5)	0.394
Length of stay—days, median (range)	8.0 (1–59)	8.0 (1–78)	0.254
Admission from medical institution—no. (%)	18 (13.1)	20 (12.7)	0.919
In-hospital mortality—no. (%)	1 (0.7)	1 (0.6)	0.923
Rate of readmission after 30 days—no. (%)	2 (1.5)	8 (4.5)	0.112
Charlson comorbidity index—median (range)	2.0 (0–8)	2.0 (0–10)	0.399
ICU/IMC—no. (%)	71 (51.8)	100 (63.7)	0.040
Severity of disease^a^ high—no. (%)	70 (52.2)	72 (49.3)	0.625
Antibiotic therapy—no. (%)	57 (41.6)	51 (32.5)	0.106
Penicillin allergy—no. (%)	13 (9.5)	8 (5.1)	0.145
Length of therapy—days, median (range)	8.0 (1–37)	7.0 (1–46)	0.774
Foreign material—no. (%)	121 (88.3)	140 (89.2)	0.818
Impaired kidney function^a^—no. (%)	29 (21.2)	36 (22.9)	0.716
Immunosuppression—no. (%)	9 (6.6)	9 (5.7)	0.765
Surgery—no. (%)	101 (73.7)	127 (80.9)	0.142
Re-surgery—no. (%)	20 (19.8)	21 (16.5)	0.523
Multidrug-resistant bacteria—no. (%)	3 (2.2)	1 (0.6)	0.342

^a^Defined as estimated glomerular filtration rate (eGFR) < 30 ml/min/1.73 m^2^

**Table 3:** Infectious diagnoses comparing P1 and P2

Courses of antibiotics	P1 (n = 66)	P2 (n = 59)	*p*-value
Infectious diagnoses—no. (%)			
Skin infection	17 (25.8)	16 (27.1)	0.863
Osteomyelitis	3 (4.5)	2 (3.4)	0.742
Bacteraemia	4 (6.1)	3 (5.1)	0.813
Shunt infection	3 (4.5)	2 (3.4)	0.742
Vascular graft infection	0 (0.0)	3 (5.1)	0.102
Urinary tract infection	14 (21.2)	6 (10.2)	0.093
Pneumonia	5 (7.6)	12 (20.3)	0.038
Intra-abdominal infection	2 (3.0)	1 (1.7)	0.626
Others	18 (27.3)	14 (23.7)	0.650

### Antibiotic consumption and changes in antibiotic prescribing

**Fig. 2:**
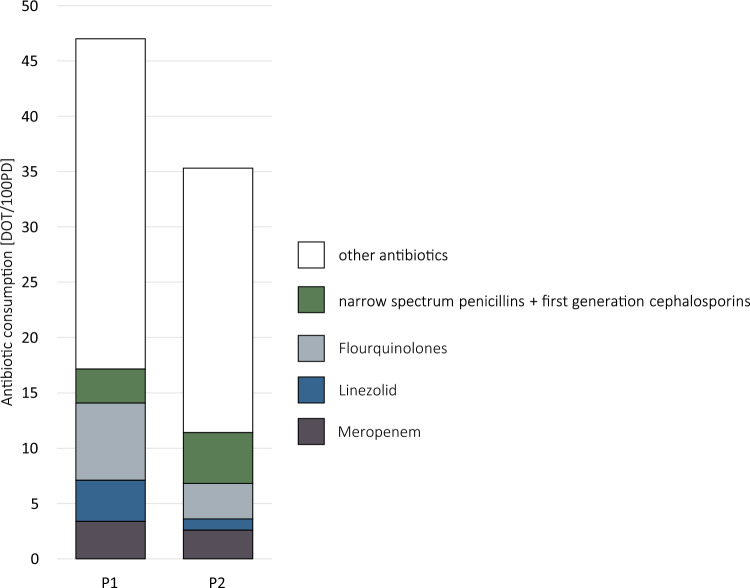
Antibiotic consumption comparing P1 and P2; DOT/100PD = days of therapy per 100 patient days

We observed a decrease in the overall consumption of antibiotics in P2, while AS interventions were provided to the ward (Fig. 2). During P1, the antibiotic consumption was 47.0 DOT/100PD which was reduced to 35.3 DOT/100PD in P2. This is a reduction of 11.7 DOT/100PD or 25% in antibiotic use.

Prescribing small spectrum antibiotics where possible is one goal of AS. Our weekly AS ward rounds combined with institutional guidelines led to a reduced consumption of the following antibiotics: Linezolid, fluoroquinolones, meropenem, cefuroxime and clindamycin were less prescribed in P2 compared to P1 (linezolid: 3.7 vs. 1.0 DOT/100PD, cefuroxime: 4.5 vs. 1.1 DOT/100PD, clindamycin: 4.6 vs. 1.9 DOT/100 PD). In contrast, the consumption of narrow spectrum penicillins and cefazolin prescribing increased by 48.4% during P2. Piperacillin/tazobactam was the most commonly prescribed antibiotic in both study periods and remained on a stable level [11.5 DOT/100 PD (P1) vs. 10.1 DOT/100 PD (P2)]. Additional data on consumption of single antibiotics are given in the supplementary material (Table SM1).

In the intervention period, there were less patients receiving antibiotic therapy compared to P1, however this was only a trend and did not reach statistical significance (41.6% (P1) vs. 32.5% (P2), *p* = 0.106). There were no changes regarding frequencies of iv-to-oral switch with 19.2% (P1) and 18.6% (P2, *p* = 0.730) in the two study periods. Likewise, the length of antibiotic therapy could only be reduced little (8 days in P1 vs. 7 days in P2) with our AS intervention. However, we observed a statistically significant higher rate of de-escalations in antibiotic therapies in the second study period: antibiotic therapies were de-escalated more often in P2 (30.5%) than in P1 (12.1%, *p* = 0.011) (Table 4). The majority of de-escalations was achieved by a change of active substance(s) to an antibiotic therapy having altogether a lower rank [5 COAs (62.5%, P1) vs. 13 COAs (72.2%, P2)]. For the remaining COAs one or more antibiotics in a combination therapy were terminated [3 COAs (37.5%, P1), 5 COAs (27.8%, P2)].

Therefore, our AS interventions significantly influenced the overall antibiotic use, the choice of substance and the de-escalation strategies.

**Table 4:** Changes in antibiotic prescribing

Courses of antibiotics	P1 (n = 66)	P2 (n = 59)	*p*-value
Route of antibiotics at beginning of			
Therapy—no. (%)			0.391
Intravenous	52 (78.8)	50 (84.7)	
Oral	14 (21.2)	9 (15.3)	
Iv-to-oral switch—no. (%)	10 (19.2)	11 (18.6)	0.730
De-escalation—no. (%)	8 (12.1)	18 (30.5)	0.011
Escalation—no. (%)	10 (15.2)	9 (15.3)	0.987

	n = 65	n = 57	
Appropriate choice of antibiotic(s)—no. (%)	45 (69.2)	45 (78.9)	0.223

	n = 46	n = 45	
Antibiotic(s) in accordance with guidelines—no. (%)	28 (60.9)	35 (77.8)	0.081

### Factors influencing antibiotic prescribing

As a next step, we evaluated if there were patient dependent factors that influenced antibiotic prescribing by physicians. For this evaluation, we compared patients with and without antibiotic therapy. Additionally, we assessed the appropriateness of the therapy as well as adherence to guidelines.

#### Comparing cases with and without antibiotic therapy

Patients receiving antibiotic therapy during their hospital stay were younger (median age 71.0 years vs. 68.0 years, *p* = 0.04). They had more comorbidities (CCMI for the group without antibiotics 2.0 vs. 3.0 for the group with antibiotics, *p* = 0.045) and the severity of their vascular disease was higher (44.1% vs. 60.0% with high disease severity, *p* = 0.073). Patients with antibiotics during their stay were more often operated on (73.1% vs. 85.2% with surgery, *p* = 0.017). Overall, this also led to a longer hospital stay in patients receiving antibiotics (7.0 days vs. 14.0 days, *p* < 0.001). Analysing the two study periods separately, more comorbidities in patients receiving antibiotics compared to those not receiving antibiotics was only shown to be significant for P2 (CCMI: 2.0 vs. 3.0, *p* = 0.005). P2 is therefore responsible for the difference shown for all patients (see above). We observed, patients receiving antibiotic therapy were re-admitted to the hospital within 30 days after discharge more often in P2 (7.8%) compared to P1 (0.0%, *p* = 0.048).

Overall, patients who were treated with antibiotics were more severely ill with the expected consequences of more surgeries and longer hospital stays. While AS interventions were provided to the ward, patients receiving antibiotic therapy suffered from more comorbidities.

#### Comparing COAs with appropriate and inappropriate antibiotic therapy

We next wanted to evaluate if the decision to treat with antibiotics was correct. For this assessment, we looked at each COA, meaning that some patients had more than one COA. Of those COAs given in both study periods, according to the retrospective evaluation the choice was correct in 90 out of 122 (73.8%) cases. In P2, we observed a higher rate of COAs rated as appropriate regarding the choice of antibiotic(s) which did not reach statistical significance, most likely due to the small sample size [69.2% (P1) vs. 78.9% (P2), *p* = 0.223]. Therefore, the initiation and choice of substance had a relatively high rate of appropriateness to begin with.

Patients with appropriate treatment displayed more comorbidities (CCMI 2.0 vs. 3.0, which failed to reach statistical significance), a slightly longer hospital stay (12.5 days vs. 15.5 days) and more revision surgeries (27.6% vs. 44.2%, also not statistically significant). A penicillin allergy reported by the patient led to more inappropriate antibiotic therapies (18.8% vs. 0.04%, *p* = 0.011). In both study periods, appropriate COAs were more often associated with impaired kidney function with an eGFR < 30 ml/min/1.73 m^2^ (P1: 5.0% (inappropriate COA) vs. 22.2% (appropriate COA), *p* = 0.087; P2: 16.7% (inappropriate COA) vs. 31.1% (appropriate COA), *p* = 0.322) which might lead to the idea that a severely impaired kidney function induces physicians to think more about the necessity of more medications.

#### Comparing COAs adherent and not adherent with the guidelines

For this assessment, only diagnoses with an existing institutional guideline could be evaluated leaving 91 COAs. Regarding the choice of substance, COAs in P2 were more often in accordance with the institutional guidelines than in P1 with a trend to statistical significance (60.9% vs. 77.8%, *p* = 0.081). Guideline adherence was seen more often in patients with more comorbidities (2.0 vs. 3.0, *p* = 0.125) and in patients with impaired kidney function (14.3% vs. 30.2%, *p* = 0.108). Only in P2, COAs in accordance with the guidelines showed a longer median length of hospital stay (9.5 d vs. 19.0 d, *p* = 0.035). For other variables including disease severity of vascular disease, presence of foreign material or immunosuppression in the respective patients, we did not find differences regarding compliance with guidelines.

## Discussion

This retrospective monocentric study demonstrates that weekly AS ward rounds combined with local antibiotic treatment guidelines improves antibiotic prescribing in a vascular surgery department. With the implementation of AS interventions, the overall antibiotic consumption decreased, as well as linezolid and fluoroquinolones consumption, without negatively affecting patient outcomes, like mortality or the rate of readmission caused by infection after 30 days. Furthermore, in the intervention period (P2), ongoing antibiotic therapy was de-escalated more often. However, our study did not find that patient dependent factors played a decisive role in the decision of antibiotic therapies. In more severely sick patients measured as more comorbidities, longer hospital stays and more impaired kidney function, we found a higher preparedness to start antibiotics and this decision was more often correct. This fact also led to a higher adherence to institutional guidelines.

A positive impact of AS programs in different surgical disciplines has previously been described in the literature [9, 14–16]. Vecchia et al. recently analysed the implementation of an ASP in a vascular surgery ward in Italy [17]. The authors—like we did—observed a decrease in carbapenem and linezolid consumption besides an increase in the rate of de-escalation of ongoing antimicrobial therapies. In contrast to our findings, Vecchia et al. noted an increase in the use of fluoroquinolones in their study. Furthermore, Bashar et al. investigated the implementation of an ASP in a vascular and a general/gastroenterology surgical ward, however, the analysis was conducted for both wards combined [18]. They observed an improvement in antibiotic consumption, quality in antibiotic prescribing and duration of antibiotic therapy. Our analysis, in contrast to Bashar et al., exclusively focused on vascular surgical patients. We could confirm their findings regarding the decrease in antibiotic consumption. In P2—the period with intense AS intervention—the COAs were more often termed appropriate and in accordance with the treatment guidelines. However, these findings did not meet statistical significance. This can be explained by the unexpected high rate of correct prescriptions in the first evaluation period on the one hand and by the relatively small sample size on the other hand.

Antibiotic therapy was more often narrowed to antibiotic(s) of lower ranking during P2. So while providing our AS interventions to the ward, an increased de-escalation rate could be achieved. De-escalation of antibiotic therapies is an important aim with regard to the development of antibiotic resistance. Schuts et al. ascertained that adherence to different AS strategies like de-escalation of antibiotic therapy even improved clinical patient outcomes like risk reduction for mortality [19]. Therefore, higher de-escalation rates are beneficial not only in terms of overall antibiotic stewardship goals, but also for the individual patient.

Shortening the duration of antibiotic therapy if possible is a main objective AS programs are aiming at. However, infections which are more often seen in vascular surgical patients like osteomyelitis or vascular graft infections require long courses of antibiotic therapy. In these cases, the task of the AS team often is to ascertain the sufficient length of therapy leading to a higher antibiotic consumption during the hospital stay.

During P2, the overall antibiotic consumption as well as the consumption of linezolid, as a last resort compound, could be distinctly reduced. The exposure to linezolid is one risk factor for linezolid resistance in *Staphylococcus epidermidis* and judicious use of linezolid is an important aim in AS [20–22]. Selection pressure through antibiotic use in general is a known driver of antibiotic resistance [23–25]. The AS interventions led to an increase of 48% in consumption of narrow-spectrum penicillins and first-generation cephalosporines. Encouraging the use of narrow-spectrum antibiotics, where possible, is one important goal of AS [25, 26].

Furthermore, the aim to reduce the use of fluoroquinolones, most of all because of possible serious adverse events, could be accomplished. This was partly because of the AS intervention. However, in October 2018, a drug safety mail announcing restrictions in use of fluoroquinolones was published. This happened during P1 and it cannot be ruled out that it might have influenced the fluoroquinolone consumption besides the conducted AS interventions. However, there was a distinct decrease in fluoroquinolone consumption in P2, which we think would hardly have happened without AS intervention provided to the ward.

Knowing patient-related factors which influence antibiotic prescribing could contribute to target future AS interventions and therefore improve the quality in antibiotic therapy for individual patients groups.

In both study periods, COAs associated with renal impairment were more likely to get classified as appropriate or to be in accordance with the local treatment guidelines. These findings were not significant but our study could have been underpowered to confirm significance. In contrast, Ingram et al. showed in their point prevalence study, that inappropriate antibiotic prescribing was associated with an elevated creatinine level [27]. This difference might be explained by the fact that we analysed the choice of antibiotics and not the correct dosing of antibiotics in patients with renal impairment as it was done by Ingram et al. One reason for our observation could be a higher physician’s awareness regarding choice of antibiotic(s) towards patients with renal impairment. Arlicot et al. also discussed the fact of higher cautiousness in dosing of antibiotics towards patients with severe renal insufficiency [28].

Anamnestic penicillin allergy was associated with a higher chance for inappropriate COAs, although the treatment guidelines contained explicit suggestions for antibiotic therapy in patients with penicillin allergy. The validity of this observation might be limited due to the very small patient numbers in this attribute. However, other studies also showed that patients with a documented penicillin-allergy in their medical history had a higher risk of being prescribed broad-spectrum antibiotics than non-allergic patients [29–31].

More comorbidities, a longer hospital stay and a (revision-)surgery leading to a higher probability of receiving antibiotic therapy should not to be discussed in detail here as it seems a consequential conclusion.

In turn, we wanted to analyse which patient related determinants are influenced by the implementation of antibiotic treatment guidelines and weekly AS ward rounds. In P2, the initiation of antibiotic therapy shifted to patients with a higher CCMI. This could lead to the conclusion that in the intervention period less patients got unnecessary antibiotic treatment. In a study conducted by Dylis et al., a higher CCMI was negatively associated with getting antibiotic treatment adherent to the guidelines [4]. This is in contrast to our findings. In P1, COAs in accordance with the guidelines were by tendency associated with a higher CCMI. The study by Alba Fernandez et al. support these findings. They compared accepted and rejected meropenem audits performed by the ASP and observed patients treated as recommended by the audit had a higher CCMI [32].

The higher CCMI in patients receiving antibiotics during P2 could also be a reason why these patients in the intervention period had more readmissions for infection after 30 days compared to P1.

This study has some limitations. It is of retrospective nature and was conducted in a single hospital. So there might be difficulties in generalizing the findings to other institutions. Furthermore, the sample size was rather small partly due to an unexpectedly low number of patients receiving antibiotics. In our opinion, this led to the fact that some of the differences did not reach statistical significance.

## Conclusion

In conclusion, we can show that once weekly AS ward rounds and an implementation of institutional antibiotic treatment guidelines improved antibiotic prescribing in vascular surgical patients significantly. A decrease in overall antibiotic consumption and consumption of linezolid and fluoroquinolones as well as an increase of narrow-spectrum beta-lactams (e.g. flucloxacillin, cefazolin) could be achieved while AS interventions were provided to the ward. After implementation of our AS interventions, we observed a shift of the initiation of antibiotic treatment towards patients with more severe illness. However, we could not identify patient dependent determinants influencing the decision of antibiotic therapies in a distinct way.

### Supplementary Information

Below is the link to the electronic supplementary material.

Supplementary file 1 (PDF 522 KB)

Supplementary file 2 (PDF 531 KB)

## Data Availability

The data presented in this study are available on request from the corresponding author.
