# Supplementary material for: The impact of antibiotic stewardship interventions and patient related factors on antibiotic prescribing in a vascular surgical department
Source: Infection. 2023 Jun 8;52(1):83–91. doi: 10.1007/s15010-023-02056-1 (PMC10810951; doi:10.1007/s15010-023-02056-1)
Supplement: Supplementary file 1 — (PDF 522 KB) [file 15010_2023_2056_MOESM1_ESM.pdf]

The impact of Antibiotic Stewardship interventions and patient related factors on antibiotic prescribing in a vascular surgical department

Gruber MM[1,2], Weber A[1,2], Jung J[1,3], Strehlau A[2], Tsilimparis N[4], Draenert R[1,]*
[1] Stabsstelle Antibiotic Stewardship, University Hospital, LMU Munich;
[2] Hospital Pharmacy, University Hospital, LMU Munich;
[3] Max von Pettenkofer Institute, Faculty of Medicine, LMU Munich;
[4] Division of vascular surgery, University Hospital, LMU Munich
*Corresponding author: rika.draenert@med.uni-muenchen.de

Table SM1 Antibiotic consumption for single substances comparing P1 and P2

	P1 [days of therapy/100 patient days]	P2 [days of therapy/100 patient days]
amoxicillin	0.0	0.0
amoxicillin/clavulanic acid	2.7	2.1
ampicillin	0.3	0.0
ampicillin/sulbactam	1.6	2.1
azithromycin	0.0	0.0
cefazolin	1.1	3.8
cefepime	1.3	0.0
cefpodoxime	0.0	0.0
ceftriaxone	0.4	0.5
cefuroxime	4.5	1.1
ciprofloxacin	6.1	2.0
clarithromycin	0.0	0.0
clindamycin	4.6	1.9
co-trimoxazole	0.2	0.8
daptomycin	0.0	0.0
doxycycline	0.0	0.9
flucloxacillin	1.4	0.4
fosfomycin	1.4	1.0
levofloxacin	0.2	0.0
linezolid	3.7	1.0
meropenem	3.4	2.6
metronidazole	0.4	0.2
moxifloxacin	0.7	1.3
benzyl penicillin	0.2	0.4
penicillin V	0.0	0.0
piperacillin/tazobactam	11.5	10.1
rifampicin	0.6	0.4
tigecycline	0.1	0.8
vancomycin	0.5	1.9
