# Supplementary material for: The impact of antibiotic stewardship interventions and patient related factors on antibiotic prescribing in a vascular surgical department
Source: Infection. 2023 Jun 8;52(1):83–91. doi: 10.1007/s15010-023-02056-1 (PMC10810951; doi:10.1007/s15010-023-02056-1)
Supplement: Supplementary file 2 — (PDF 531 KB) [file 15010_2023_2056_MOESM2_ESM.pdf]

The impact of Antibiotic Stewardship interventions and patient related factors on antibiotic prescribing in a vascular surgical department

Gruber MM[1,2], Weber A[1,2], Jung J[1,3], Strehlau A[2], Tsilimparis N[4], Draenert R[1,]*
[1] Stabsstelle Antibiotic Stewardship, University Hospital, LMU Munich;
[2] Hospital Pharmacy, University Hospital, LMU Munich;
[3] Max von Pettenkofer Institute, Faculty of Medicine, LMU Munich;
[4] Division of vascular surgery, University Hospital, LMU Munich
*Corresponding author: rika.draenert@med.uni-muenchen.de

Figure SM1 Institutional antimicrobial treatment guidelines

SOP ANTIBIOTIKATHERAPIE GEFÄßCHIRURGIE (VERSION 2.0)

PERIOPERATIVE ANTIBIOTIKAPROPHYLAXE (PAP)<br>(gültig für primären Eingriff sowie Revisionseingriffe)	
**Häufigste Erreger**	→ S. aureus<br>→ S. epidermidis und andere koagulase-negative Staphylokokken<br>→ Gram-negative Erreger: sehr selten

**Eingriff**	**Standard**	**Penicillinallergie:** bei Anaphylaxie!
**kein Fremdmaterial**	**Keine PAP**	**Keine PAP**
**Implantation von Fremdmaterial oder Revisionseingriff mit Fremdmaterial in situ**	**Cefuroxim** 1,5 g i.v.<br><br>**single shot**	**Clindamycin** 600mg i.v.<br><br>**single shot**
**Bei vorbestehender Infektsituation im OP-Gebiet (z.B. Ulcus, Wundinfekt) Fortführung der bestehenden antibiotischen Therapie!**		

Bei **MRSA** (z.B. Besiedelung/positives Screening zum Zeitpunkt der OP/Wundinfekt mit MRSA): zusätzliche Gabe von Vancomycin 1000mg single shot über 1,5 h (bei Patienten > 90 kg Vancomycin 1500 mg über mind. 2,5 h)

Verabreichung der PAP:
30 – 60 min vor Hautschnitt.
Bei Vancomycin: 60 – 120 min vor Hautschnitt – Verabreichung in der Regel bereits auf Station bzw. bei Abruf.

Zweite Dosis PAP:

Substanz	Halbwertszeit	Wiederholte Gabe
**Cefuroxim**	1 – 2 h	3 h
**Clindamycin**	2 – 4 h	6 h
**Vancomycin**	4 – 8 h	-
**Wiederholte Gabe bei Blutverlust > 1L**		

Häufige gefäßchirurgische Infektionen

Vor Therapiebeginn
→ **Mikrobiologische Diagnostik**
→ **Cave:** Vorbefunde aus der Mikrobiologie beachten
→ **Anpassung der Dosis an Nierenfunktion bedenken**

Mikrobiologische Diagnostik:
1) Bei Fieber, schweren systemischen Entzündungszeichen, Verdacht auf Sepsis etc. → mind. 2 Blutkultursets abnehmen
2) **Mikrobiologische Proben aus dem Infektionsgebiet:**
Wertigkeit: Gewebeproben > Aspirat > Abstrich
Einsendung:
- **Gewebeprobe:** in Abstrichröhrchen oder sterilem Gefäß mit NaCl oder Ringer bedeckt, **kein** Formalin
- **Aspirat:** Nativ in sterilem Gefäß einsenden ggf. zusätzlich Blutkulturflaschen beimpfen (insbesondere wenn die Zeit bis zur Verarbeitung der Probe verlängert ist z.B. Nacht /Wochenende und wenn ausreichend Material vorhanden ist)
- **Abstrich:** möglichst tief am Wundgrund abstreichen und in Röhrchen verbringen

Probe auf rosa B-Schein genau und leserlich bezeichnen (z.B. „Wundheilungsstörung linke Leiste mit freiliegendem Kunststoffbypass" statt „linke Leiste"). Untersuchung routinemäßig auf: Entzündungs- und Eitererreger, Anaerobier (Anzukreuzen auf rosa B-Schein). Bei Blutkultur: Uhrzeit der Abnahme angeben.

**Trockene Nekrosen**	Keine mikrobiologische Diagnostik
	**Keine antibiotische Therapie**
**Chronische Wunden/Ulcera bei pAVK/ diabetischem Fußsyndrom**	Keine Routinediagnostik, da Besiedelung i.d.R. irrelevant. Mikrobiologische Diagnostik nur durchführen, wenn Infektion vermutet wird.
	**Wenn möglich, Therapie erst nach Erregernachweis beginnen!**<br><br>Falls nicht möglich:<br><br>**Moderat** (lokale Entzündungszeichen):<br>**Ampicillin/Sulbactam 4 x 3g i.v.**<br>Alternative bei Penicillinallergie:<br>Moxifloxacin 1 x 400mg i.v./p.o.<br><br>**Schwer** (systemische Entzündungszeichen):<br>**Piperacillin/Tazobactam 3 x 4,5g i.v.**<br>Alternative bei Penicillinallergie:<br>Moxifloxacin 1 x 400mg i.v. + Metronidazol 3 x 500mg i.v.<br><br>**Bei plausiblem Erregernachweis: Therapie anpassen!**<br><br>Bei septischem Schock o. Sepsis: siehe Sepsis SOP

**Osteomyelitis/ Osteitis**	Knochenbiopsie bzw. -resektate bevorzugen (vor allem im Rahmen von Amputationen).
	**Wenn möglich, Therapie erst nach Erregernachweis beginnen.**<br><br>Falls nicht möglich:<br>**Piperacillin/Tazobactam 3 x 4,5g i.v. + Clindamycin 4 x 600mg i.v.**<br><br>**Bei plausiblem Erregernachweis: Therapie anpassen!**<br><br>**Therapiedauer:**<br>Bei Amputation/erfolgreicher chirurgischer Sanierung: 14 d<br>Ansonsten 6 Wochen (evtl. länger, abhängig von Klinik und Bildgebung). Nach 1 Woche, bei gutem klinischen Ansprechen, Umstellung auf orale Therapie bedenken.
**Gefäßprothesen -infektion**	**Vor Therapiebeginn immer mikrobiologische Diagnostik veranlassen!**<br><br>Abnahme von Blutkulturen (mind. 3 Sets)!<br><br>Nach Therapiebeginn: bevorzugt Prothesenmaterial einschicken. Falls nicht möglich periprothetisches Gewebe, Aspirat oder intraoperativer Abstrich.<br><br>Untersuchung routinemäßig auf: Entzündungs- und Eitererreger, Anaerobier und Candida (Anzukreuzen auf rosa B-Schein).<br><br>Zusätzlich Uni-PCR anfordern (Freitext auf rosa B Schein)
	**Initialtherapie: Vancomycin** nach Dosierungsempfehlung<br><br>Gegebenenfalls in Kombination bei fehlendem Ansprechen oder Sepsis:<br><br>**β-Laktam:**<br>Piperacillin/Tazobactam 3 x 4,5g i.v. oder Meropenem 3 x 1g i.v.<br><br>**Antimykotikum:**<br>Caspofungin 1 x 70mg i.v. (wenn Gewicht unter 80kg ab Tag 2: 1 x 50mg)<br><br>**Bei plausiblem Erregernachweis: Therapie anpassen!**<br><br>**Therapiedauer:**<br>nach Klinik, Sanierungsmöglichkeit und Erreger; jedoch grundsätzlich lange Therapiedauer.

**Postoperative Wundinfektion**	Mikrobiologische Diagnostik vor Beginn der antibiotischen Therapie und erneut vor geplantem Sekundärverschluss.
	Wenn **ambulante Therapie** möglich:<br>**Amoxicillin/Clavulansäure 2 x 875/125mg p.o.**<br>Alternative bei Penicillinallergie + Wunde außerhalb Leistengegend: Clindamycin 3 x 600mg p.o.<br>Alternative bei Penicillinallergie + Wunde in Leistengegend: Moxifloxacin 1 x 400mg p.o.<br><br>Wenn **parenterale Therapie** erforderlich:<br>**Ampicillin/Sulbactam 3 x 3g i.v.**<br>Alternative bei Penicillinallergie + Wunde außerhalb Leistengegend: Cefuroxim 3 x 1,5g i.v. + Clindamycin 3 x 600mg i.v.<br>Alternative bei Penicillinallergie + Wunde in Leistengegend: Moxifloxacin 1 x 400mg i.v. + Clindamycin 3 x 600mg i.v.<br><br>**Bei plausiblem Erregernachweis: Therapie anpassen!**<br><br>**Therapiedauer:**<br>unkompliziert: 5 – 7 Tage<br>sonst nach Klinik und Erreger
**Staphylococcus-aureus-Bakteriämie (StauB)**	**Def.:** Wachstum von S. aureus in einer oder mehreren Blutkulturen<br>**→ Bei Nachweis von S. aureus in der BK besteht immer Handlungsbedarf!**<br>**Bei unkomplizierter StauB:**<br>**Flucloxacillin 4-6 x 2g i.v.**<br>oder<br>**Cefazolin 3-4 x 2 g i.v.**<br><br>**Bei komplizierter StauB oder MRSA**<br>sowie für **Diagnostik und weitere Informationen** siehe kliniküGbergreifende SOP
**Deeskalation**	Nach Erreger und Antibiogramm

Seltene Infektionen in der Gefäßchirurgie	
Hautinfektionen	s. klinikübergreifende SOP
Harnwegsinfekt	s. klinikübergreifende SOP
Pilzinfektionen	Infektiologisches Konsil
Pneumonien (CAP/HAP/ Aspirations-pneumonie)	s. klinikübergreifende SOP
Sepsis	Therapie richtet sich nach dem mutmaßlichen Fokus, von dem die Sepsis ausgeht.<br><br>Sepsis ohne Fokus:<br>→ Piperacillin/Tazobactam 4 x 4,5g i.v.<br>→ bei MRGN-Risiko:<br>Meropenem 3 x 2g i.v.<br>→ bei dringlichem V.a. MRSA:<br>+ Vancomycin nach Dosierungsempfehlung oder<br>Daptomycin 1x 8mg/kgKG i.v.<br>(Daptomycin nicht bei V.a. Pneumonie)<br><br>s. klinikübergreifende SOP

Literatur, Referenzen:
1) Venkatesan, Aradhana M., et al. "Practice guideline for adult antibiotic prophylaxis during vascular and interventional radiology procedures." Journal of Vascular and Interventional Radiology 21.11 (2010): 1611-1630.
2) Bratzler, Dale W., et al. "Clinical practice guidelines for antimicrobial prophylaxis in surgery." Surgical infections 14.1 (2013): 73-156.
3) Suerbaum, Sebastian, et al., eds. Medizinische Mikrobiologie und Infektiologie. Springer-Verlag, 2016.
4) Li, Ho-Kwong, et al. "Oral versus intravenous antibiotics for bone and joint infection." New England Journal of Medicine 380.5 (2019): 425-436.
5) Li, Hai Lei, Yiu Che Chan, and Stephen W. Cheng. "Current evidence on management of aortic stent-graft infection: a systematic review and meta-analysis." Annals of vascular surgery 51 (2018): 306-313.

Erstellung durch: **ABS-Team** (M. Gruber, Dr. A. Weber, Dr. J. Jung, Prof. Dr. R. Draenert)
Klinische Mikrobiologie und Krankenhaushygiene (Dr. B. Grabein)
Gefäßchirurgische Klinik (M. Götz, Prof. Dr. N. Tsilimparis)
Max von Pettenkofer-Institut (Dr. J. Jung)

Version 2.0 – Stand August 2019
